# Using a 5G network in hospitals to reduce nosocomial infection during the COVID-19 pandemic

**DOI:** 10.1038/s43856-022-00118-3

**Published:** 2022-05-12

**Authors:** Li Wen, Zhiwen Ou, Wenzhou Duan, Weijie Zhu, Xiongzhi Xiao, Ying Zhang, Huanquan Luo, Weibin Cheng, Wanmin Lian

**Affiliations:** 1 Information Department, Guangdong Second Provincial General Hospital, Guangzhou, China; 2 Institute for Healthcare Artificial Intelligence Application, Guangdong Second Provincial General Hospital, Guangzhou, China

**Keywords:** Epidemiology, Viral infection

## Abstract

Wen et al. discuss how implementing a 5G network in hospitals can be used to reduce nosocomial infections. Such systems can reduce the spread of COVID-19.

## Teaser

The COVID-19 pandemic has resulted in nosocomial transmission of COVID-19 within hospitals and other healthcare settings such as residential homes and primary care settings. Here, we discuss how a 5G network can be used to reduce such infections.

Nosocomial infections are those that are acquired in a healthcare setting and they have been commonly reported during the coronavirus disease 2019 (COVID-19) pandemic. For example, 48 COVID-19 cases, including 28 healthcare workers (HCWs), 13 patients, and 7 accompanying persons, were associated with a nosocomial outbreak in the pediatric dialysis unit of the University Hospital of Münster^1^. Although the percentage of the at-risk population who contract severe acute respiratory syndrome coronavirus 2 (SARS-CoV-2; attack rate) is variable during a nosocomial outbreak, it can be as high as 60%, resulting in high mortality^2^. In addition to the impact on patients, HCWs have been reported to have at least a tenfold increased risk of being infected with SARS-CoV-2 compared with the general population^3^. Infection of HCWs and the associated mortality have detrimental effects on healthcare service as well as the morale of front-line staff^4^. There are also increased risks associated with spreading the virus to patients with co-morbidities who are more vulnerable to the effects of the virus^5^.

The Guangdong Second Provincial General Hospital (GD2H, Guangzhou, Guangdong Province, China) recently built the first 5G-powered smart hospital in China in conjunction with Huawei^6^. The 5G technology included in the hospital features a computer network able to process a very high volume of wireless data with minimal delay, resulting in a fast and efficient transfer of information. Several features of the technology have the potential to reduce the occurrence of nosocomial infections (Table 1). In this article, we highlight examples of how the 5G technology has enabled practices and behavior within the hospital to be modified to reduce the likelihood of nosocomial infections.

**Table 1:** The key information technology components that might impact nosocomial infections.

Reduced in-person visits	Outpatient services	Ward management	Hand hygiene monitoring	Management of medical waste
AI doctor	Registration	Virtual electronic fence	Hand hygiene awareness	Recovery
Online triage	Charging	VR remote visit		Storage
Teleconsultation	Waiting	VR remote ward round		Classification
Electronic prescription	Specimen collection	Robot disinfection	Handwashing compliance	Packaging
Drug distribution	Examination	Robot drug delivery		Transportation

*AI* artificial intelligence, *VR* virtual reality.

## Reduced in-person visits

During the pandemic, a large number of people have been to the hospital, resulting in significant logistical challenges for hospitals and healthcare professionals. GD2H uses artificial intelligence (AI) and 5G technology to provide disease diagnosis under the supervision of clinicians (AI doctors). The high bandwidth and wireless nature of 5G also enable high-definition real-time online consultations, psychological counseling, and other online services to be provided^7^. Electronic prescriptions can be issued and AI can also be used for epidemiological investigations, intelligent screening of COVID-19 symptoms, and pre-hospital triage. These AI systems use automated screening algorithms that were designed using a decision tree that classified patients according to their symptoms, travel history, and exposure to COVID-19. From June 30, 2020 to June 30, 2021, 89,607 online consultations were made and 17,033 electronic prescriptions were issued. By offering these online options, in-person visits are reduced, decreasing the risk of nosocomial infection in patients and medical staff.

## Outpatient services

Outpatient services in hospitals are a key component of many diagnosis and treatment pathways. The notice on improving the Prevention and Control of Infection in Fever Outpatient and Medical Institutions issued by the Chinese National Health Commission stated that “three areas and two channels” must be established to deal with outpatients, requiring separation of areas into polluted, semi-polluted and clean areas, plus separate channels for medical personnel and patients^8^. GD2H set up outpatient services for patients with a fever in strict accordance with these requirements, providing a one-stop service area integrating registration, payment, waiting, specimen collection, examination, and other functions, such as consulting-related services. This enables patients to complete the whole visit, from registration to examination, in a single area. Test results and reports are transmitted to patients’ smartphones in real time using the app DingBei Doctor, developed by GD2H. From June 30, 2020 to June 30, 2021, 243,694 people registered on the hospital’s WeChat public platform, and 962,464 payments were made. This reduces the movement of patients within the hospital and the number of trips required to the hospital, reducing contact opportunities between patients in high-risk areas.

## Ward management

Effective management and protection of inpatients is also required to reduce nosocomial infections^9^. GD2H uses semi-active Radio Frequency Identification technology, which makes objects and personnel uniquely identifiable as virtual representations in an internet-like structure (the Internet of Things). This enables a virtual electronic fence to be established around a pre-set security area (inpatient activity area). When patients leave the security area set by the system, or unauthorized persons enter the area, the system alarm is triggered. In parallel, the whereabouts of personnel in the hospital can be traced using face recognition software. A virtual reality (VR) system enables a patient’s family to visit remotely. When the patient’s family enters the visiting room, the doctor informs the ward nurse who puts a smart trolley at the bedside of the patient being visited. The smart trolley is equipped with camera, computer, microphone, and speaker. The family can wear VR glasses to watch the patient in the ward, and the doctor can explain the patient’s condition to the family simultaneously. Medical staff can also use mobile phones to undertake remote ward rounds at any time and anywhere using the VR system. This minimizes contact between doctors, patients, and their families within the ward.

AI-driven robots or AI-assisted equipment further reduce contact between patients and hospital staff, reducing the direct exposure of medical personnel to highly infectious environments^10^. For example, GD2H uses robot disinfection and drug delivery in high-risk areas such as isolation wards and wards containing patients with COVID-19. A disinfection robot is a disinfection machine that integrates ultraviolet disinfection, ultra-dry fog hydrogen peroxide sterilization, and air filtration. Using the 5G network and laser navigation technology, the robot moves autonomously according to the set route, and automatically carries out regular and thorough disinfection in complex environments, ensuring all areas are appropriately disinfected. The drug delivery robot automatically arrives at the bedside of the patient according to the bed number set by the nurse. A smart health watch worn by the patient ensures each patient can only access their medicine.

## Hand hygiene monitoring

The hands of medical staff are an important transmission route of nosocomial infection^11^. Handwashing is the simplest, most effective, and most economical way to control nosocomial infection^12^. GD2H uses AI and Ultra Wide Band (UWB) technology to monitor hand hygiene. UWB is a wireless technology that allows indoor positioning of people or objects with accuracy reaching the decimeter level^13^. AI technology removes interference from noisy data and improves the accuracy of identification. When medical staff enters the handwashing area from the infected area, the system reminds the user to wash their hands and tracks handwashing behavior to improve hand hygiene awareness and monitor handwashing compliance.

## Management of medical waste

**Fig. 1:**
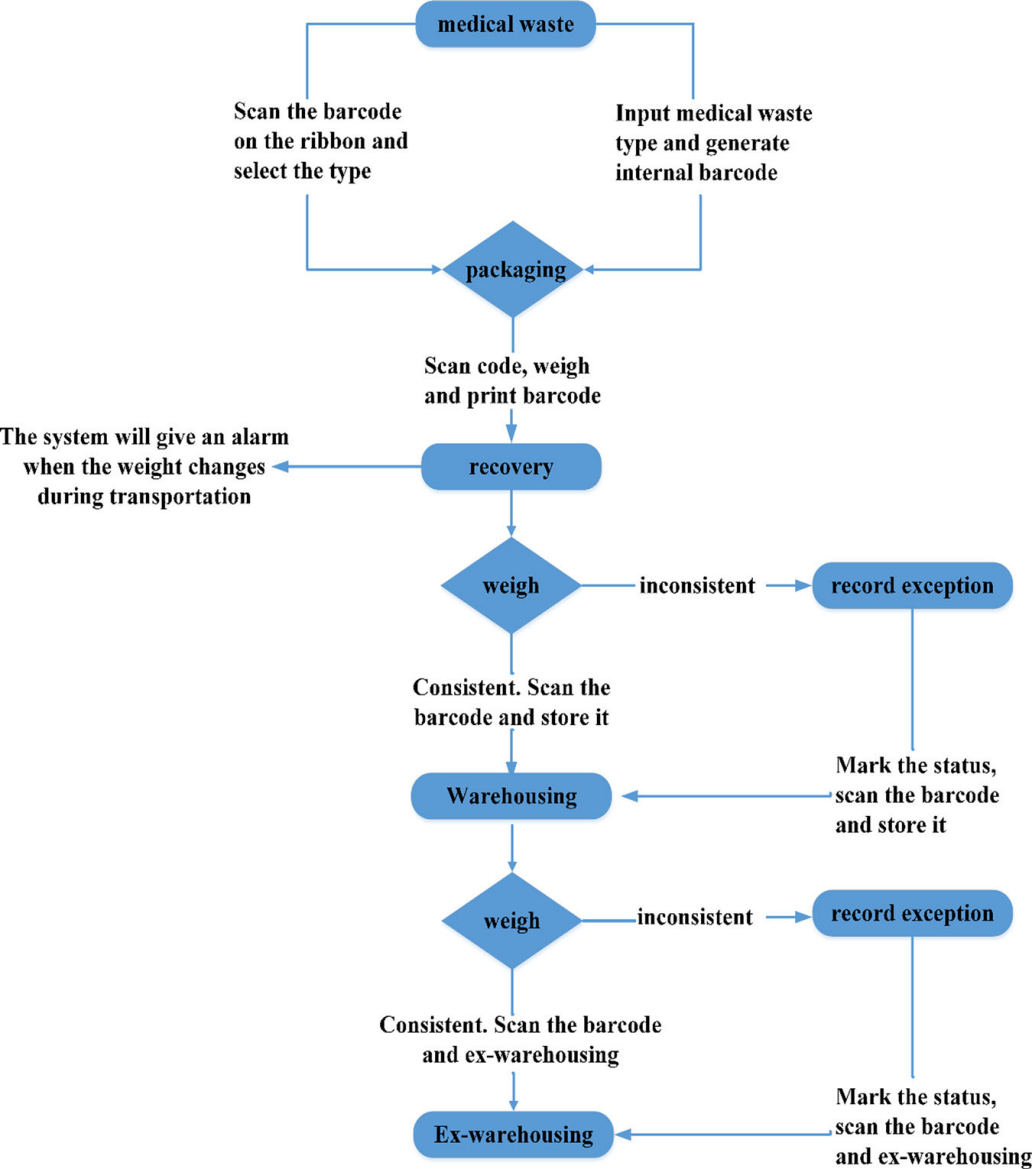
The workflow followed by the medical hazardous waste management system.

During the COVID-19 outbreak, demand for personal protective equipment and medical supplies increased dramatically. Proper disposal of medical waste reduces the potential risk of pathogen transmission among medical personnel, patients, and the public^14^. The GD2H medical hazardous waste management system monitors the recovery, storage, classification, and packaging of medical waste in real time. This system combines an Internet of Things application platform with intelligent identification and network technology. It supports single-item packaging for toxic and harmful radioactive medical waste and tracks the whole process of waste disposal. This ensures different types of waste are disposed of appropriately (Fig. 1).

## Concluding remarks

By utilizing 5G, AI, and the Internet of Things, GD2H has implemented early intervention, active monitoring, and whole-process tracking to reduce the potential risk of nosocomial infection and better control any occurrences. Whilst these technologies were implemented prior to the COVID 19 pandemic, the pandemic has enabled their utility to be demonstrated. Using hospital monitoring data, it was found that as of June 30, 2020, GD2H, as a provincial emergency hospital, had admitted 52 confirmed cases of COVID-19, >1300 suspected cases, and had been visited by patients with fever >68,000 times, during which time no nosocomial infection was detected. In contrast, a news report from the Health Times reported that nosocomial infection occurred in >10 hospitals lacking this technology across China including in Beijing, Hebei, Heilongjiang, Liaoning, and Shandong^15^. We hypothesize that the absence of nosocomial infections at GD2H was due to the implementation of the technologies we describe here.

There are challenges to integrating modern technology into hospitals, including training, the dependence of the HCWs on the system, data security, and privacy protection^7^. Nevertheless, we anticipate that the use of 5G and AI in conjunction with the Internet of Things will enable wider control of nosocomial infection as well as improved monitoring, tracking, and faster feedback of any wider impact of such infections. In the future, it is hoped that 5G technology will be further popularized and AI algorithms will be optimized to promote the integration of information technology into medical practice.
